# Genome mining and metabolic profiling of the rhizosphere bacterium *Pseudomonas* sp. SH-C52 for antimicrobial compounds

**DOI:** 10.3389/fmicb.2015.00693

**Published:** 2015-07-07

**Authors:** Menno Van Der Voort, Harold J. G. Meijer, Yvonne Schmidt, Jeramie Watrous, Ester Dekkers, Rodrigo Mendes, Pieter C. Dorrestein, Harald Gross, Jos M. Raaijmakers

**Affiliations:** ^1^Laboratory of Phytopathology, Wageningen UniversityWageningen, Netherlands; ^2^Institute for Pharmaceutical Biology, University of BonnBonn, Germany; ^3^Departments of Pharmacology and Chemistry and Biochemistry, Skaggs School of Pharmacy and Pharmaceutical Sciences, University of California, San DiegoSan Diego, CA, USA; ^4^Brazilian Agricultural Research Corporation, Embrapa EnvironmentJaguariuna, Brazil; ^5^Department of Pharmaceutical Biology, Pharmaceutical Institute, University of TübingenTübingen, Germany; ^6^Department of Microbial Ecology, Netherlands Institute of Ecology (NIOO-KNAW)Wageningen, Netherlands

**Keywords:** beneficial microbes, rhizosphere bacteria, antimicrobial peptides, Pseudomonads, genome sequencing, mass spectrometry, biocontrol

## Abstract

The plant microbiome represents an enormous untapped resource for discovering novel genes and bioactive compounds. Previously, we isolated *Pseudomonas* sp. SH-C52 from the rhizosphere of sugar beet plants grown in a soil suppressive to the fungal pathogen *Rhizoctonia solani* and showed that its antifungal activity is, in part, attributed to the production of the chlorinated 9-amino-acid lipopeptide thanamycin (Mendes et al., 2011). To get more insight into its biosynthetic repertoire, the genome of *Pseudomonas* sp. SH-C52 was sequenced and subjected to *in silico*, mutational and functional analyses. The sequencing revealed a genome size of 6.3 Mb and 5579 predicted ORFs. Phylogenetic analysis placed strain SH-C52 within the *Pseudomonas corrugata* clade. *In silico* analysis for secondary metabolites revealed a total of six non-ribosomal peptide synthetase (NRPS) gene clusters, including the two previously described NRPS clusters for thanamycin and the 2-amino acid antibacterial lipopeptide brabantamide. Here we show that thanamycin also has activity against an array of other fungi and that brabantamide A exhibits anti-oomycete activity and affects phospholipases of the late blight pathogen *Phytophthora infestans*. Most notably, mass spectrometry led to the discovery of a third lipopeptide, designated thanapeptin, with a 22-amino-acid peptide moiety. Seven structural variants of thanapeptin were found with varying degrees of activity against *P. infestans*. Of the remaining four NRPS clusters, one was predicted to encode for yet another and unknown lipopeptide with a predicted peptide moiety of 8-amino acids. Collectively, these results show an enormous metabolic potential for *Pseudomonas* sp. SH-C52, with at least three structurally diverse lipopeptides, each with a different antimicrobial activity spectrum.

## Introduction

*Pseudomonas* species are ubiquitous in aquatic and terrestrial habitats, and are intensively studied for their abilities to promote plant growth and to suppress plant pathogens (Weller, 2007; Berendsen et al., 2012). The main mechanisms involved in plant pathogen control are induced systemic resistance, competition, antibiosis and parasitism (Van Loon et al., 1998; Haas and Defago, 2005; Van Wees et al., 2008; Lugtenberg and Kamilova, 2009). To date, a wide variety of bioactive compounds involved in pathogen control have been identified for Pseudomonads (Gross and Loper, 2009; Raaijmakers et al., 2010; Raaijmakers and Mazzola, 2012). These include siderophores, hydrogen cyanide, 2,4-diacetylphloroglucinol, pyrrolnitrin, pyoluteorin, phenazines, 2,5-dialkylresorcinol, quinolones, gluconic acid, rhamnolipids, and various structurally diverse lipopeptides (Gross and Loper, 2009; D'Aes et al., 2010).

Comparative genomics studies of *Pseudomonas* species have shown substantial diversity between the genera, between species, and even between strains belonging to the same species (Silby et al., 2011; Wu et al., 2011; Loper et al., 2012; Redondo-Nieto et al., 2012). Loper et al. (2012) further revealed that most of the genes encoding bioactive compounds map outside the core genome. This includes genes encoding the Non-Ribosomal Peptide Synthetases (NRPSs). NRPSs possess a modular structure and each module is a building block for the stepwise incorporation of an amino acid in the peptide moiety (Gross and Loper, 2009; Marahiel and Essen, 2009). NRPSs are responsible for the production of an array of antimicrobial compounds including lipopeptides (LPs). Intriguing features of LPs are their enormous structural diversity and diverse natural roles in microbial behavior (Raaijmakers and Mazzola, 2012). By the expansion of available genome sequences also the number of identified NRPS gene clusters has increased considerably over the past years. To exploit the hidden genetic and metabolic potential in genome sequences, a number of search tools and approaches have been developed, including regulator-based discovery (Hassan et al., 2010), metabolic networking, peptidogenomics and advanced mass spectrometry methods (Kersten et al., 2011; Watrous et al., 2012). This also led to the discovery of NRPS gene clusters involved in the production of structurally novel LPs (Liu et al., 2014).

Recently, we discovered *Pseudomonas* sp. SH-C52, a strain representative of a larger population of Pseudomonads that contributes to the natural suppressiveness of a soil against the fungal plant pathogen *Rhizoctonia solani* (Mendes et al., 2011). The gene cluster responsible for the activity of strain SH-C52 against *R. solani* is encoded by an NRPS, predicted to synthesize a 9-amino-acid chlorinated LP, designated thanamycin (Mendes et al., 2011). The production and partial structure of thanamycin was resolved by live colony mass spectrometry (Watrous et al., 2012). Next to thanamycin, strain SH-C52 was also found to produce a set of 2-amino-acid LPs, designated brabantamides A-C, which contain a glycosylated 3-hydroxy fatty acid tail. Brabantamide A displays activity against Gram-positive bacteria, including *Staphylococcus aureus* and *Arthrobacter crystallopoietes* (Reder-Christ et al., 2012; Schmidt et al., 2014). The 12-kb gene cluster for brabantamide biosynthesis includes the NRPS gene (*braB*), a glycosyltransferase (*braA*) and a specific FAD-dependent Baeyer–Villiger monooxygenase gene (*braC*). Biosynthesis of brabantamides is complex: a linear di-peptide is formed by BraB after which the sugar moiety is attached by the glycosyltransferase BraA. This glycosylated di-peptide is subsequently rearranged by the brabantamide-specific monooxygenase BraC (Schmidt et al., 2014).

To extend our knowledge on the biosynthetic repertoire of bioactive compounds of plant-associated *Pseudomonas* sp. SH-C52, we sequenced the genome and performed detailed *in silico* as well as metabolomic analyses. Here we show that genome-based phylogeny places strain SH-C52 in the *Pseudomonas corrugata* subgroup of the *P. fluorescens* clade. Next to the known thanamycin and brabantamide gene clusters, *in silico* analysis revealed four additional NRPS gene clusters. We further characterized the antimicrobial activity spectrum of thanamycin and brabantamide and identified the structure, activity and gene cluster of a novel LP with a 22-amino acid peptide moiety designated thanapeptin.

## Materials and methods

### Strains and growth conditions

The bacterial strains *Pseudomonas* sp. SH-C52 (Mendes et al., 2011), derivatives from this strain, and *Pseudomonas syringae* pv. syringae B728a were grown on Pseudomonas agar F (PSA, Difco) or in King's medium B (KB, King et al., 1954). *Bacillus megaterium* and *Pectobacterium atrosepticum* SCRI1043 were cultured on Luria Bertani (LB) agar plates or in LB broth. When needed, growth media were supplemented with 25 μg/ml gentamycin 100 μg/ml kanamycin, and/or 25 μg/ml tetracycline. All bacteria were grown at 25°C.

Mutants in the thanapeptin gene cluster were obtained by screening the transposon insertion mutant library of *Pseudomonas* sp. SH-C52 for mutants that showed loss of or reduced activity against *R. solani* (Mendes et al., 2011). The initial screening showed the thanapeptin gene cluster mutants (Tn-*tnpA* and Tn-*tnpC*) to have a very minor reduction in the growth-inhibitory activity against *R. solani* (data not shown). The site-directed mutants in the thanamycin gene cluster KO25 and KO26 were obtained previously and were designated in this manuscript as dThaB and dThaC2, respectively. All fungi and oomycetes used in this study were cultured on Potato Dextrose Agar (PDA, Difco, Becton, Dickinson and Company, USA), except for *Phytophthora infestans* strain 88069 and *Phytophthora capsici* LT3239 which were cultured on Rye Sucrose Medium and on V8 medium (Latijnhouwers et al., 2004), respectively. *P. infestans* was cultured at 18°C; whereas all other fungi and oomycetes were cultured at 25°C.

### Illumina genome sequencing and assembly

Illumina sequencing was performed by BGI (China) according to their protocols. For this purpose, two libraries with insert sizes of 0.5 and ~2 kb were constructed and sequenced. In order to ensure the accuracy of follow-up analysis, several steps were performed to filter the raw data: removal of reads with a certain proportion of Ns' bases or low complexity reads (10% as default); removal of reads with a certain proportion of low quality (≤Q20) bases (40 bases as default); removal of adapter contamination (15 bp overlap between adapter and reads as default); removal of contamination due to duplication. For reads with low sequence quality additional processing was performed, i.e., removal of reads with significant poly-A structure, and removal of reads with a k-mer frequency of 1. During the processing 16% of the original read data was eliminated. Short reads were assembled into genomic sequences using SOAPdenovo, a BGI developed assembler (Li et al., 2010). Using mapping information gaps were filled and single base pairs were proofread. The usage rate of reads was obtained according to read mapping, from which the genome coverage was estimated.

### Additional sequencing, sequence adjustments, and gene annotation

Sequence information on the thanamycin gene cluster from strain SH-C52 (Mendes et al., 2011) was incorporated in the genome sequence to close gaps in the Illumina acquired assembly. In addition, for the predicted 22 amino-acid NRPS gene cluster, gaps were closed by additional standard Sanger sequencing (Macrogen, Amsterdam). Specific primer pairs were designed for PCR to cover the gaps present in the original Illumina assembly (Table S1). Primers were used for both PCR and sequencing. PCR products were sequenced in both orientations. Gene annotation was performed on the complemented genome sequence by use of RAST (http://rast.nmpdr.org) (Overbeek et al., 2014). The genome sequence and gene annotation are available at the NCBI and EMBL database (accession number CBLV000000000).

### Genome analysis and comparisons

Genome comparisons were performed by use of nucleotide BLAST (BLASTN) in BioEdit (Ibis Biosciences). Genes identified by Loper et al. (2012) in species from the *Pseudomonas fluorescens* clade were used for BLASTN analysis on the SH-C52 genome sequence. For *Pseudomonas corrugata* CFBP5454 (ATKI00000000) and *Pseudomonas mandelii* JR-1 (NZ_CP005960) these analyses were performed online at the NCBI database. In general, genes were considered to be present when more than 65% identity with the reference sequence(s), and more than 85% of coverage was observed. In addition to the BLAST analysis, secretion systems were identified by screening gene annotations.

A whole-genome phylogenetic analysis was performed with the genomes of species of the *P. fluorescens* group as reported by Redondo-Nieto et al. (2012), with the addition of the genomes of *Pseudomonas corrugata* CFBP5454 (ATKI00000000) and *P. mediterranea* CFBP5447 (AUPB00000000). Phylogenetic trees were built by a Composition Vector approach using the web server CVTree with a *k*-value of 6 (Xu and Hao, 2009). Trees were generated by the Neighbor joining algorithm with *P. aeruginosa* PAO1 as the outgroup. Phylogenetic trees were visualized by MEGA5.1 (MEGA).

For the identification of secondary metabolite gene clusters the genome sequence was analyzed by both the on-line analysis programs NP.searcher (Li et al., 2009) and antiSMASH (Blin et al., 2013).

### Thanapeptin gene cluster analysis

The gene cluster identified to encode a NRPS of 22 amino acids, was further analyzed for the domains in the synthetases by the PKS/NRPS predictor (Bachmann and Ravel, 2009). Moreover, the Adenylation (A)-domains predicted by the PKS/NRPS predictor were analyzed by a phylogenetic comparison to known A-domains. MEGA5.1 (MEGA) was used for alignments and subsequent tree construction, using the neighbor-joining method and 500 bootstrap replicates. The combination of the predictions by NP.searcher, antiSMASH, PKS/NRPS predictor and phylogenetic analysis led to a consensus prediction presented in **Figure 5**. The first Condensation (C1)-domain predicted for the thanapeptin gene cluster was also subjected to phylogenetic analysis (as described above), with C1-domains of other NRPS gene clusters, to compare the thanapeptin C1-domain to cyclic and non-cyclic (lipo)peptides NRPSs (De Bruijn et al., 2007).

### *In vitro* inhibition assays

For *in vitro* growth inhibition of fungi and oomycetes, SH-C52 and its derivative strains were, using an overnight culture in KB, spot-inoculated (3 μl) at the periphery of 1/5th strength PDA (pH 7.0) plates. After incubation for 2 days at 25°C, an agar plug (4-mm-diameter) from a freshly grown plate with the target organism was transferred to the center of the 1/5th PDA plate. Inhibition of radial growth of the fungus or the oomycete was monitored from 2 days after incubation up to 2 weeks after incubation, depending on the growth speed of the target organism. For *in vitro* bacteria and yeast inhibition assays, bacteria and yeast cells were included, after autoclaving and cooling to 45°C, into the 1/5th PDA medium at a concentration of ~10^5^ cfu/ml. After solidifying and sufficient drying of the plates, SH-C52 and its derivative strains were, using an overnight culture in KB, spot-inoculated (3 μl) at the periphery of the bacteria or yeast containing plates.

### Activity of brabantamide A against *phytophthora infestans*

To test both the brabantamide A antimicrobial effect and the effect on phospholipase activity of *P. infestans*, individual *P. infestans* mycelial plugs were transferred to a 24-wells plate (Greiner Bio-One) with each well containing 2 ml rye sucrose broth (Latijnhouwers et al., 2002). Brabantamide A was added to the broth at the concentrations indicated. Brabantamide A was dissolved in DMSO, and DMSO concentrations were normalized for all wells to a final concentration of 1% (v/v). Growth of *P. infestans* was monitored for 6–8 days at an incubation temperature of 18°C. Mycelial mass was determined after drying the mycelium for 2 days at 60°C. Growth experiments were performed twice, each with two replicates per treatment. As similar results were obtained, representative results of one experiment are shown.

To assay the effect of brabantamide A on *P. infestans* phospholipid metabolism, mycelial plugs were labeled overnight with 10 μCi carrier free ^32^PO^3−^_4_ (GE Healthcare, Diegem, Belgium). Brabantamide A treatments were performed simultaneously with the labeling or for 15 min after the labeling. All treatments contained a final concentration of 1% (v/v) DMSO and 0.1% (v/v) of n-butanol. The latter was well below phospholipid metabolism stimulatory concentrations (Latijnhouwers et al., 2002) and was included to detect transient PLD activity (Munnik et al., 1995; Meijer et al., 2011). Incubations were halted by addition of perchloric acid (final concentration 5%) and subsequent freezing in liquid nitrogen. After thawing 3.75 volume of CHCl_3_/CH_3_OH/1M HCl (50:100:1 by vol) and two glass beads (Ø = 3 mm) were added and the samples were again frozen in liquid nitrogen. Samples were thawed and thereafter vigorously shaken for 30 min. Samples were further treated and analyzed as described previously (Latijnhouwers et al., 2002). Radiolabeled phospholipids separated by TLC were detected and quantified by phospho-imaging (Storm, Molecular Dynamics; Sunnyvale, CA, USA).

### Mass spectrometry analysis

For NanoDESI experiments the instrument setup was according to Watrous et al. (2012). The nanoDESI source coupled to a Thermo LTQ-FT-ICR MS capable of collision-induced dissociation. All analyses were performed in positive ion mode in the mass range of *m/z* from 200 to 2000. Both the primary and the nanospray capillaries were 150 μm o.d. × 50 μm i.d., with solvent being delivered and removed from the liquid bridge at approximate 45° angles. The solvent used was acetonitrile/0.05% formic acid in water (1:1) running at a flow rate of 0.8–2.5 μl/min. The droplet size using this configuration was ~200 μm in diameter. MALDI imaging was performed according to Liu et al. (2010). For both the MALDI imaging and the NanoDESI experiments, the *Pseudomonas* sp. SH-C52 and its derivatives were grown on 1/5th PDA plates.

For antimicrobial assays and detailed MS analysis of the thanapeptin derivatives, strain *Pseudomonas* sp. SH-C52 was pre-grown overnight in 5 ml LB at 28°C. This overnight culture was used to streak-inoculate (10-μl loop) ISP2 agar plates [~12 ml/plate, 4 g/l yeast extract (Sigma), 4 g/l dextrose (Sigma), 10 g/l malt extract (Sigma), 7.5 g/l agar (Sigma), 7.5 g/l agar (Teknova)]. Streak-inoculated plates were grown at 28°C for 36–44 h. Thanapeptin was extracted by scraping cells from the plates into solvent (60% acetonitrile/40% water/0.1% formic acid). Extractions were performed by shaking for 1 h. The cell extract was transferred to 50-ml centrifuge tubes and cells were spun down. The extraction was performed on the same cells for a total of 3 times. The supernatant was dried by rotovap and the pellet was retrieved by rinsing three times of 6 ml of the extraction solvent. The extraction fluid was collected in a centrifuge tube and spun down again. Subsequently, the supernatant was dried by rotovap again using a small 20-ml scintillation vial until dry. The vial was then rinsed by three washings with 400 μl of extraction solvent and placed in a 1.5 ml centrifuge tube and centrifuged at 14,000 rpm. The supernatant was injected in the HPLC containing a C18 4.6 × 150 mm 5 μm analytical column with a 250 μl sample loop. Mobile phase A was 100% water with 0.1% formic acid. Mobile phase B was 100% acetonitrile with 0.1% formic acid. The flow rate was set to 1.0 ml/min and a gradient elution was used with 15% mobile phase B ramped to 95% mobile phase B over 45 min. The eluent was collected using a fraction collector set to collect 1 ml fractions. Fractions were checked for purity and the right mass using MALDI (Bruker). Purified thanapeptin and its derivatives were subsequently used in antimicrobial assays, and assayed by MS analysis by a Thermo LTQ-FT-ICR MS capable of collision-induced dissociation.

### Thanapeptin activities

Compounds were tested in interaction with target organisms by different techniques. To test activity against bacteria and the yeast *Rhodoturula pilimanae*, plates containing the target strain were used. Bacteria and yeast were added at a concentration of ~10^5^ cfu/ml to the 1/5th PDA medium. After sufficient drying of the plate, filter discs with the test compounds at indicated concentrations were added to the plate. Plates were incubated at 25°C and growth was monitored for 4 days. Activity against fungal and oomycete strains was tested both in liquid and on plate. For plate assays, thanapeptin derivatives at a concentration of 50 μM were added directly at the border of a 1/5th PDA, after which an agar plug (4 mm diameter) from a freshly grown plate with the target fungal or oomycete strain was positioned in the center of the plate. Hyphal growth was monitored from 2 days after incubation up to 2 weeks after incubation, dependent on the growth speed. Testing of compound activity against fungal and oomycete strains in liquid culture was performed by addition of the compound to 1 ml of liquid 1/5th PDB in a 12 wells culture plate (Greiner Bio-One), in which an agar plug (4 mm diameter) from a freshly grown plate with the target organism was submerged. Growth experiments were incubated at 25°C, except for *P. infestans*, which was cultured at 18°C.

## Results

### General genome features and phylogeny

**Figure 1:**
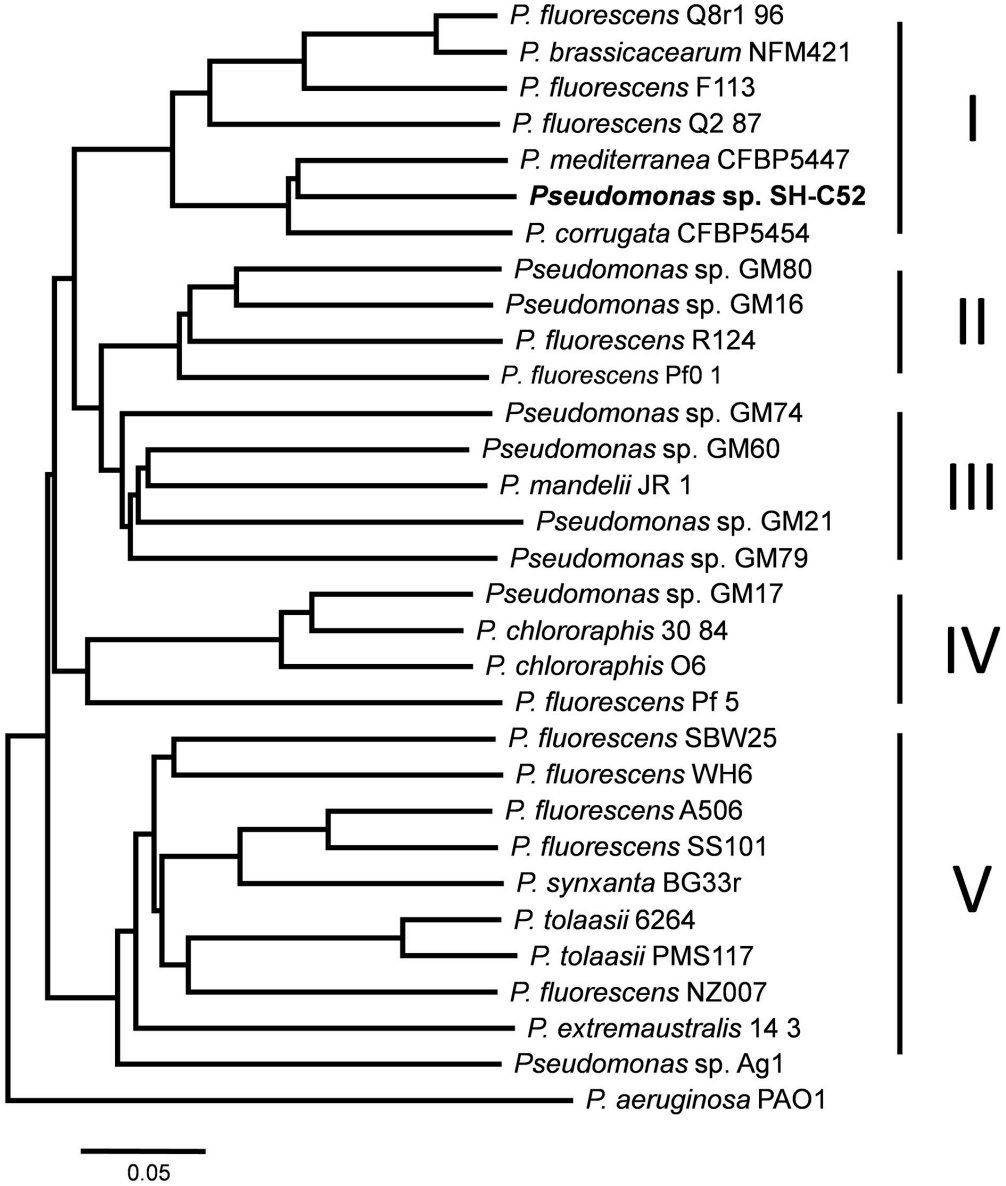
**Whole genome phylogenetic analysis of ***Pseudomonas*** sp. SH-C52**. Phylogenetic tree constructed by use of CVtree, and based on the analysis performed previously by Redondo-Nieto et al. (2012), with the addition of strains SH-C52, *P. corrugata* CFBP5454 and *P. mediterranea* CFBP5447.

Sequencing and assembly showed that the genome size of *Pseudomonas* sp. SH-C52 is ~6.3 Mb with a GC content of 61.0%. The genome was assembled in 596 contigs and 25 scaffolds, with an estimated coverage of 99.2%. For annotation, 384 contigs of at least 100-bp and covering 6.3 MB were used. In total, 5579 ORFs were annotated with 5523 CDSs and 56 tRNAs (Table 1). Previous analysis of the 16S rRNA sequence placed SH-C52 within the *P. fluorescens* clade (Mendes et al., 2011). At that time, however, no conclusive species designation was obtained. Expanding the former genome-wide phylogenetic analysis on *P. fluorescens* species (Redondo-Nieto et al., 2012) with the SH-C52 genome and the recent draft genomes of *P. corrugata* CFBP5454 and *P. mediteranea* CFBP5447, showed that strain SH-C52 clusters within subgroup I of the *P. fluorescens* group. Within subgroup I, SH-C52 and the strains of *P. corrugata* and *P. mediterranea* form a separate clade (Figure 1).

**Table 1:** **General genome sequence information of *Pseudomonas* sp. SH-C52**.

	**Scaffold**	**Contig > 100 bp**
Number	25	384
Total Length (bp)	6,356,481	63,12,590
Max length (bp)	1,572,034	292,658
Min length (bp)	505	102
Sequence GC(%)	61.0	61.0
Gene number	5808	5579
tRNAs	55	56

### *In silico* analysis of *pseudomonas* sp. SH-C52 primary metabolism

The metabolic potential of *Pseudomonas* sp. SH-C52 was analyzed by comparing genes from primary metabolism with those known for the *P. fluorescens* clade (Loper et al., 2012). In this analysis, also its close relative *P. corrugata* strain CFBP5454 was included as well as *P. mandelii* strain JR-1 as a representative strain for subgroup III. The comparative analysis showed that for several of the metabolic activities in species of the *P. fluorescens* clade, the gene sequences are also found in strain SH-C52. These include genes involved in catabolism of L-arabinose, mannitol, myo-inositol, and ethanol, as well as genes coding for gelatinase and lipase. The ethanol catabolism genes, however, were only detected in five of the 12 other studied strains of the *P. fluorescens* clade, including subgroup I strains Q8r1-96, Q2-87, and *P. corrugata* CFBP5454. In contrast, genes encoding catabolic pathways for trehalose (10 strains), levan sucrase (7 strains) and L-tryptophan (7 strains, only subgroup IV and V) are present in multiple strains of the *P. fluorescens* clade, but were not detected in the SH-C52 genome (Table 2). Interestingly, comparing the SH-C52 genes with genes in the genome of *P. corrugata* CFBP5454 showed a few remarkable differences. Genes for trehalose catabolism were found in *P. corrugata* but not in SH-C52. The same was found for the nitrate reductase gene cluster previously reported for *P. corrugata* strains (Siverio et al., 1993). These differences in the presence of genes involved in primary metabolism further strengthen the phylogenetic delineation (Figure 1) where SH-C52 is closely related to, but different from *P. corrugata*.

**Table 2:** **Primary metabolism and corresponding genes of *Pseudomonas* SH-C52**.

**Function**	**I**				**II**	**III**	**IV**			**V**			
	**SH-C52**	**Pc 5454**	**Q8r1-96**	**Q2-87**	**Pf0–1**	**Pm JR-1**	**Pf-5**	**30–84**	**O6**	**SBW25**	**A506**	**SS101**	**BG33R**
Gelatinase	BN844_0247	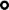	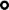	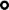	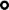	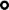	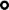	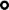	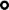	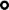	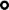	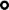	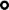
Lipase	BN844_0271	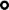	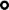	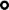	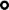	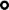	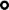	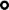	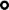	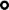	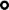	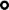	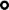
Phenylacetic acid							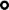	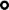	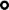				
Sodium Benzoate			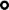		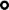	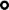	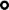	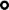	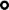			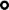	
Trehalose		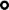	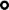		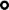	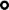	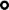	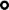	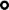	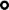	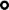	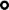	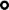
Levan sucrase			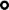	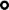				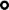	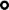	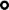	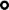	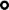	
L-arabinose	BN844_3151-3149	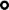	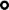	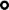	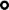	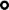		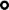	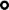	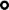	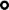	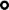	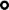
Nitrate reduction		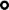	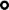	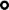		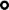			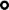				
D-serine								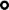	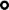				
Denitrification		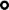	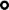	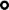		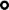							
Ethanol	BN844_0564	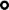	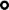	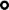		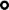	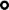						
Sorbitol			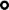	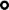		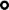				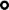	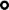	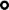	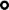
Mannitol	BN844_0168	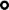	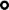	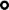	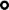	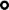	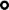	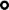	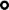	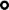	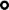	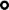	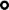
myo-Inositol	BN844_0814	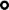	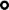	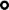		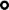	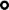	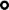	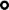	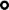	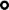	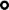	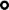
D-xylose	BN844_0249	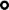	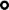	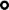	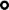	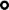				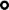	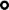	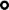	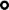
L-tryptophan							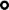	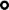	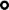	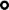	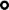	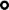	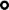

^*^Functions are based on the 10 strains studied by Loper et al. (2012). The roman numbers are indicating subgroups within the P. fluorescens clade as identified by Redondo-Nieto et al. (2012), for which also the gray shades are indicative. Strains studied by Loper et al. (2012) are also included in the table, with the addition of P. corrugata CFBP5454 (Pc 5454) and P. mandelii JR-1 (Subgroup III). For SH-C52 the locus tags for the corresponding genes are given.

### Genome analysis of traits involved in plant-microbe interactions

*In silico* analysis of the SH-C52 genome for genes involved in the production of phytohormones, volatiles, and plant signaling compounds led to the identification of putative pathways for acetoin/butanediol and for GABA catabolism (Table 3). Also the different bacterial secretion systems are of crucial importance for plant-microbe interactions (Korotkov et al., 2012; Russell et al., 2014; Tampakaki, 2014). The genome of SH-C52 contains two gene clusters encoding type II secretion systems with significant sequence similarity (>70%) to the Xcp and Hxc systems of *P. aeruginosa* (Table 3). Genes encoding potential substrates of the type II secretion system, such as lipases, proteases, esterases and alkaline phosphatases (Douzi et al., 2012) are well presented in the SH-C52 genome (Tables 2, 3). No genes encoding a type III secretion system were found in the SH-C52 genome, yet two putative effector genes for this secretion system were identified (BN844_1916 and BN844_3678). Type VI secretion systems are conserved among Gram-negative bacteria and are thought to be involved in bacteria-bacteria interactions (Russell et al., 2014). Two gene clusters were found to encode type VI secretion systems of which one (BN844_4205-4227) was typed as a HSI-I locus and the second (BN844_0760-0774) as a HSI-III locus (Table 3).

**Table 3:** **Secondary metabolite production and corresponding genes of *Pseudomonas* sp. SH-C52**.

		**I**				**II**	**III**	**IV**			**V**			
		**SH-C52**	**Pc 5454**	**Q8r1-96**	**Q2-87**	**Pf0-1**	**Pm JR-1**	**Pf-5**	**30-84**	**O6**	**SBW25**	**A506**	**SS101**	**BG33R**
	**ANTIBIOTICS**													
	DAPG			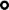	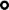			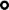						
	HCN	BN844_0823-0821	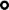	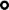	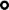	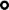	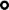	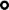	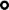	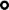				
	Phenazine								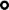	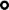				
	Pyrrolnitrin							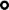	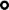	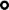				
	Rhizoxin							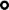						
	Pyoluteorin							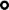						
	HPR								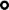	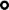				
	**NRPS/SIDEROPHORE/TOXINS**													
	CLP^*^	**Figure 2**	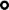			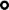		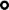			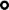		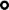	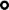
Siderophore	Pyoverdine			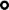	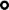	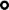	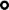	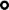	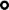	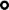	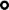	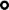	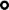	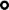
	Pyochelin							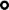						
	Pseudomonine											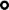		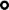
	Achromobactin	BN844_0513-0523	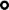						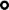	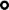				
	Hemophore							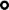			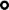		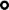	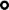
	NRPS1—MgoA	BN844_4882	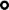	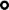	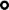	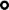	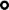		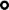	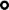	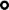	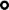	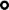	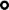
	NRPS3	BN844_2194-2167^#^	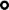	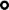							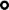			
	Bacteriocin	BN844_3382-3419	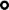	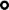	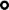	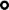	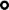	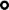	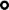	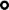	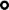	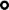	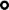	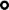
Insect toxins	FitD toxin					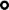		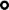	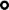	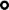				
	Tcc1											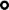	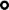	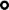
	Tcc2	BN844_3853-3849	2	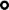	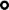									
	Tcc3													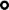
	Tcc4	BN844_2985-2986	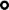	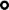		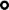								
	Tcc5						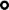			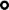				
	**EXOENZYMES**													
	Chitinase 1^^*^^					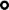	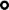		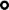	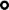		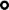	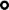	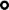
	Chitinase 2							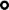	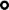	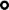				
	AprA	BN844_0247	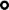	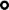	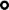	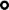	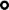	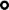	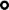	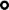	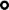	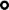	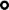	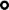
	AprX							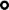	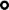	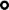				
	Protease 1											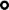	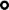	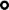
	Protease 2												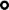	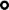
	Protease 3											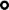		
	Protease 4													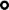
	Pectate lyase										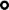			
	**SECRETION SYSTEMS**													
Type II	T2SS Xcp^*^	BN844_4825-4833	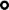		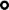				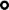	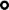				
	T2SS Hxc			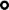	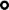	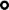		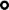	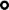	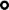	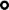			
	T2SS Hxc-2	BN844_2047-2057									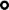			
	T2SS novel						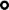				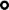	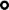	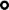	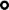
Type III	T3SS 1			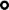	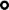									
	T3SS 2										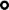	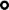	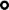	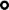
	T3SS 3				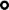									
T3SS effectors	ExoU				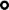						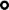	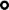	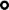	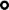
	RopB			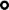	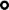									
	RopM			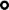	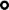									
	RopAA-1			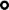	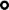									
	Novel Effector				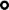									
	Putative Effectors	BN844_1916, _3678	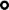								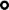	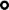	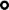	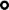
Type VI	HSI-I	BN844_4205-4227	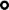	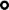	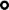			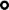	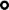	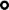	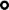	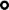	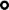	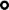
	HSI-II		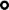		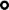	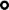	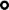		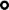	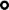		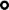	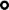	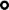
	HSI-III	BN844_0760-0774	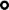	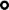	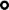	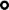								
	TSS4									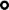				
	**PLANT COMMUNICATION**													
	IAA biosynthesis								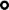	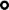				
	IAA catabolism													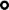
	PAA catabolism							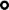	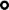	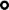				
	ACC deaminase			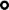										
	Butanediol synthesis							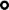	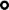	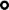				
	Acetoin/Butanediol^*^	BN844_0610-0604	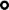	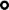	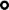			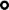	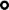					
	Acetoin catabolism											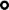	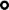	
	GABA catabolism	BN844_4573-4574	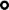	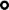	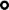	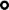	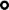	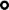	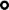	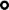	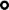	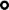	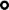	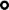

**Figure 2:**
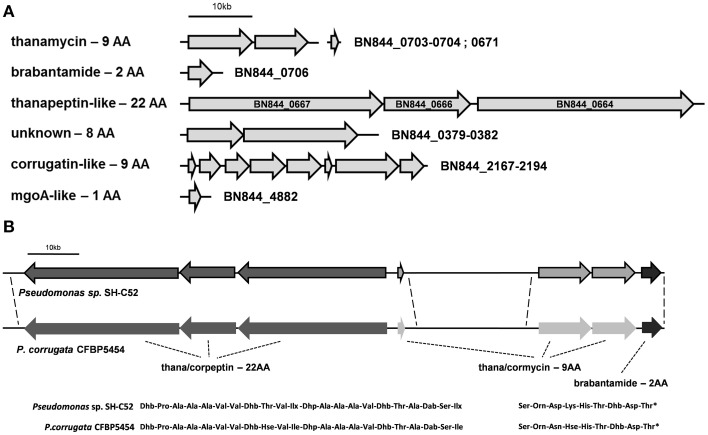
**Non-ribosomal peptide synthetase gene clusters present in the genome of ***Pseudomonas*** sp. SH-C52**. **(A)** The six NRPS gene clusters predicted in the genome of SH-C52. **(B)** A comparison of the SH-C52 thanamycin-thanapeptin-brabantamide cluster with the putative equivalent cluster of *P. corrugata* CFBP5454. Thana/corpeptin related NRPS genes are in dark gray, thana/cormycin NRPS genes are in light gray, and brabantamide genes are in near black. The *P. corrugata* genes are not bordered, as the genome information is scattered over contigs and needs to be confirmed. Dotted lines indicate (expected) sequence conservation. Amino acids are indicated in their standard three-letter annotation. Non-standard amino acids were abbreviated as follows: Hse, homoserine; Ilx, isoleucine or leucine; Dab, 2,4-diaminobutanoic acid; Dhb, 2,3-dehydro- 2-aminobutanoic acid; Dhp, dehydro-2-aminopropanoic acid (dehydroalanine). The asterisk in the thana/cormycin structure indicates chlorination of this amino acid.

* *Functions are based on the 10 strains studied by Loper et al. (2012). The roman numbers are indicating subgroups within the P. fluorescens clade as identified by Redondo-Nieto et al. (2012), for which also the gray colors are indicative. Strains studied by Loper et al. (2012) are also included in the table, with the addition of P. corrugata CFBP5454 (Pc 5454) and P. mandelii JR-1 (Subgroup III). For SH-C52, the locus tags for the corresponding genes are given. Cyclic lipopeptides (CLP) for SH-C52 are discussed in the text and listed in **Figure 2***.

# *The SH-C52 NRPS3-corrugatin gene cluster sequence is incomplete and divided over different contigs*.

### Genome analysis for secondary metabolites

To predict putative secondary metabolites, such as antibiotics, siderophores and other NRPS genes, the genome sequence was first screened by BLAST for ORFs of known compounds produced by other species and strains of the *P. fluorescens* clade (Loper et al., 2012). Only five SH-C52 clusters corresponded with known clusters (Table 3). First of all, the gene cluster for hydrogen cyanide production (HCN, BN844_0823-0821) was found in the SH-C52 genome and corresponded to the gene cluster found in eight out of the 10 previously sequenced *Pseudomonas* species (Loper et al., 2012). No pyoverdine synthetase gene cluster was found in SH-C52, which was also the case for *P. corrugata*. This corresponds to the observation that *P. corrugata* strains are known to be pyoverdine negative (Meyer et al., 2002). The siderophore gene cluster identified for SH-C52 is achromobactin (BN844_0513-0523). This gene cluster is not common among members of the *P. fluorescens* species clade, but has been described for *P. syringae* strains (Berti and Thomas, 2009; Owen and Ackerley, 2011) and for two *P. chlororaphis* strains (Loper et al., 2012), and is also found in the genome of *P. corrugata* strain CFBP5454 (WP_024777665-670, Table 3). In addition to the achromobactin gene cluster, an NRPS gene cluster (BN844_2194-2167) was identified with similarity to genes presumably involved in ornicorrugatin biosynthesis. The putative (orni)corrugatin gene cluster identified in SH-C52 was also found in *P. fluorescens* strains Q8r1-96 and SBW25. Ornicorrugatin and the related compound corrugatin are so-called secondary siderophores (Matthijs et al., 2008). For *P. fluorescens*, however, conclusive experiments that link the production of (orni-) corrugatin to its putative gene cluster are still lacking.

Two other gene clusters in SH-C52 were predicted to encode insecticidal toxins. The first gene cluster is related to the Tcc2 toxin for which the genes are also found in the genomes of *P. fluorescens* strains Q8r1-96 and Q2-87. The second insect toxin gene cluster is similar to that of Tcc4 toxin (Table 3). No bacteriocin gene clusters could be identified with similarity to those found in other genomes of the *P. fluorescens* clade. However, a prophage gene cluster (BN844_3382-3419) flanked by *cinA* and *mutS*, typical for prophages present in other *Pseudomonas* genomes (Loper et al., 2012), was identified in the genome of SH-C52.

### Non-ribosomal peptide synthetases

The SH-C52 genome contains six NRPS gene clusters, including the putative (orni-) corrugatin gene cluster (Figure 2). The other five clusters include the known NRPS gene clusters for the synthesis of thanamycin (BN844_0670-0673 and BN844_0703-0704, Mendes et al., 2011), and brabantamide (BN844_0705-0707, Schmidt et al., 2014). In close proximity to the thanamycin and brabantamide gene clusters, a third NRPS gene cluster (BN844_0667-0664) was identified (Figure 2). This NRPS is predicted to code for a 22 amino-acid lipopeptide with similarity to corpeptin (Figure 2) produced by *P. corrugata*. Therefore, in agreement with the naming of cormycin and corpeptin, we designated the predicted 22-amino-acid compound of SH-C52 as thanapeptin. The genome of *P. corrugata* CFBP5454 contains several NRPS genes with similarity to the 22 amino-acid related gene cluster in SH-C52. However, in *P. corrugata* this gene cluster is divided over different contigs. Recently, a partial gene cluster containing a partial NRPS gene, encoding two adenylation-domains and two transport genes, were linked to corpeptin production (Strano et al., 2014). Because of the incomplete nature of the draft genome sequence for strain CFBP5454, it remains unclear if the organization of the gene clusters for cormycin and corpeptin of *P. corrugata* is also similar to that of thanamycin and thanapeptin in SH-C52. However, consistent with the gene organization in SH-C52, a putative brabantamide-like gene cluster was also found in *P. corrugata* down-stream of the putative cormycin gene cluster (Figure 2). In addition, the biocontrol strain *P. fluorescens* In5 was recently reported to produce the antagonistic metabolites nunamycin and nunapeptin, also with similarity to cormycin and corpeptin, respectively. Although gene clusters for nunamycin and nunapeptin were presented (Michelsen et al., 2015), the genome information of strain In5 was not provided.

The fourth NRPS gene cluster (BN844_0379-0382) found in SH-C52 is predicted to code for an NRPS that produces an 8-amino-acid lipopeptide, with no significant similarity to other known compounds in general and dedicated databases such as NCBI and the Norine peptide database. This gene cluster appears to be unique for SH-C52, as it was not found in other genomes. The last NRPS gene cluster identified in SH-C52 shows similarity to *mgoA*-like gene clusters, encoding only one adenylation domain. MgoA or MgoA-regulated compound(s) were proposed to regulate the expression of pathogenicity factors in *P. entomophila* and *P. syringae* (Vallet-Gely et al., 2010; Carrion et al., 2014), although the underlying mechanism is yet unknown.

### Thanamycin: gene cluster and antimicrobial activity

**Figure 3:**
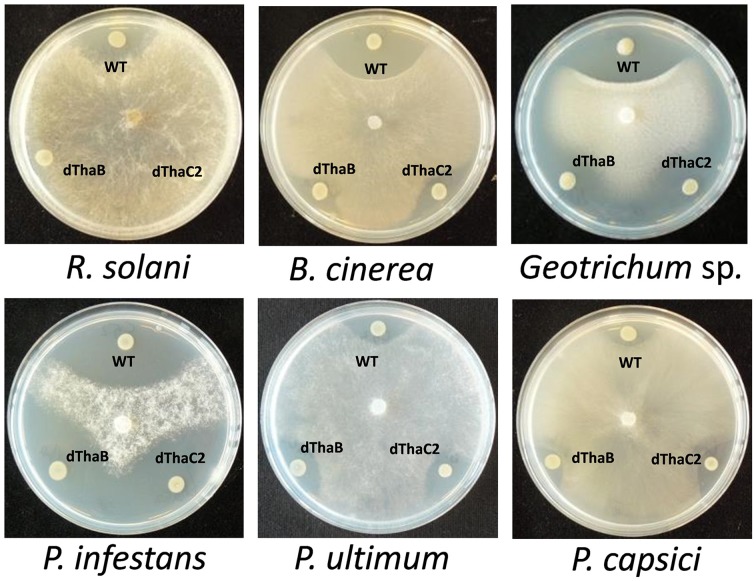
**Antagonistic activity of ***Pseudomonas*** sp. SH-C52**. Wild-type (WT) and the thanamycin mutant strains (ThaB, ThaC2) were tested in dual culture assays for activity against fungal plant pathogens **(upper panel)** and oomycete plant pathogens **(lower panel)**.

Based on mutant analysis, the thanamycin gene cluster was shown to be important for the growth-inhibitory activity of strain SH-C52 against *Rhizoctonia solani* (Mendes et al., 2011). The thanamycin gene cluster shows similarity to a fragmented NRPS gene cluster in *P. corrugata* CFBP5454 (Figure 2), but the incomplete and scattered *P. corrugata* sequences complicate a clear comparison between the thanamycin and cormycin gene clusters. Alignments of the thanamycin NRPSs and the annotated parts of cormycin NRPSs show 85–97% protein identity. To test the antimicrobial activity-spectrum of thanamycin, wild type SH-C52 and two thanamycin biosynthesis mutants (Mendes et al., 2011) were tested for activity against fungi, oomycetes and bacteria. Here, we show that the activity spectrum of thanamycin is not exclusive for *R. solani*, but extends to an array of other fungi (Figure 3) and the Gram-positive bacterium *Bacillus megaterium* (data not shown). Also cormycin has been shown to be active against *B. megaterium* and the yeast *Rhodotorula pilimanae* (Scaloni et al., 2004). In contrast, thanamycin has little activity against oomycete pathogens (Figure 3) and the Gram-negative bacteria *Pseudomonas syringae* and *Pectobacterium atrosepticum* (data not shown).

### Brabantamide: gene cluster and antimicrobial activity

The gene cluster and the brabantamide compounds were previously identified (Schmidt et al., 2014). Interestingly, a putative brabantamide gene cluster is also present in the genome of *P. corrugata* (Figure 2) as well as in the genome of *P. fluorescens* DSM 11579 (Johnston et al., 2013).

**Figure 4:**
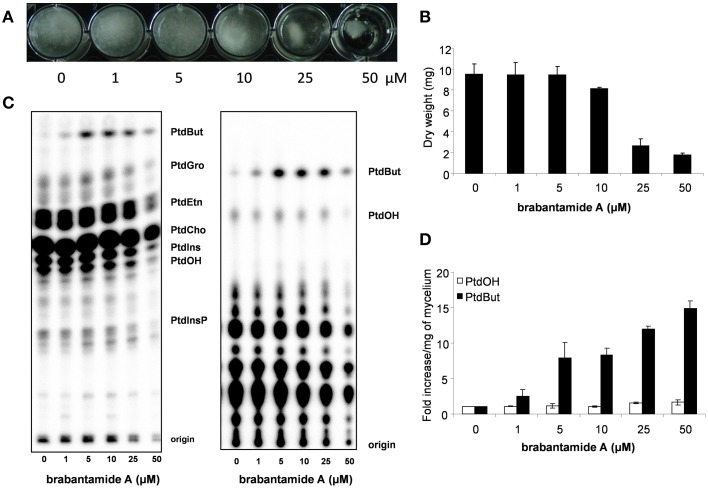
**Antimicrobial and phospholipase D stimulating activity of brabantamide A in interaction with ***Phytophthora infestans*****. **(A)** Growth of *P. infestans* in liquid medium with different concentrations of brabantamide A. **(B)** Dry weight of *P. infestans* grown in liquid medium (*n* = 2). **(C)** Lipids extracted from *P. infestans*, separated by alkaline (left) or ethylactetate (EtAc) TLC (right) and analyzed by phosphoimaging. The origin of the chromatogram, phosphatidylinositolphosphate (PtdInsP), phosphatidic acid (PtdOH), phosphatidylinositol (PtdIns), phosphatidylcholine (PtdCho), phosphatidylethanolamine (PtdEtn), phosphatidylglycerol (PtdGro) and phosphatidylbutanol (PtdBut) are indicated. **(D)** PtdOH and PtdBut were quantified from the EtAc TLC and presented as the fold increase when compared to control conditions (*n* = 2). Error bars represent standard deviations.

In previous studies, brabantamides were shown to have activity against specific Gram-positive bacteria (Reder-Christ et al., 2012; Schmidt et al., 2014). Here, we show that brabantamide A, at a concentration of 50 μM, has activity against the oomycete plant pathogens *Phytophthora capsici, Pythium ultimum*, and *P. infestans* (Figure 4). No antifungal activity was observed at this concentration (data not shown). For *P. infestans*, mycelial growth was already inhibited at concentrations above 5 μM resulting in a less dense mycelial mat (Figure 4A). Previous studies suggested that structural analogs of brabantamide A inhibit phospholipase A2 of rabbit (Thirkettle et al., 2000). When [^32^P]Pi-labeled hyphae of *P. infestans* were incubated for 15 min with brabantamide, no significant variation in the phospholipid levels was observed (data not shown). However, overnight exposure of *P. infestans* mycelium to brabantamide A led to an increase in the phosphatidylbutanol (PtdBut) levels. This already occurred at a concentration of 1 μM brabantamide A, the lowest concentration tested (Figure 4). Other phospholipid levels were not affected based on quantification. The enhanced PtdBut levels indicates a stimulatory effect of brabantamide A on phospholipase D activity. In contrast, no indication was found for the inhibition of phospholipase A2 in this assay.

### Thanapeptin: gene cluster, structural analysis and antimicrobial activity

**Figure 5:**
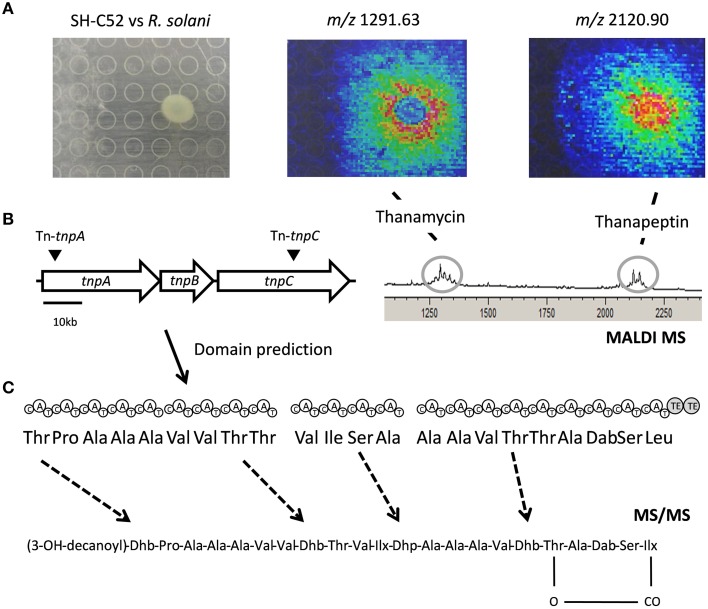
**Thanapeptin genetic and chemical analysis. (A)** The upper panel shows the interaction of SH-C52 in interaction with *R. solani*, as imaged by MALDI imaging mass spectrometry (IMS). Thanamycin and thanapeptin detection by MALDI IMS are shown. **(B)** The middle panel presents the thanapeptin gene cluster with the position of the transposon insertions, and the modules encoded by the gene clusters and the prediction for the amino acids assembled. On the middle left, the partial spectrometry profile of the MALDI IMS of the SH-C52 and *R. solani* interaction is shown. **(C)** At the bottom the tentative thanapeptin structure obtained from tandem mass spectrometry data is presented. Dashed arrows indicate amino acids that are difficult to predict from genome information, and that needed to be resolved by mass spectrometry.

The thanapeptin gene cluster consists of three NRPS genes. BLAST analysis of the protein sequences showed similarity with the NRPSs for the production of the LP syringopeptin of *P. syringae*. Indeed, the first gene of the thanapeptin cluster starts with a specific condensation (C)-domain, a so-called C1 starter domain (data not shown), suggesting *N*-acylation of the first amino acid in the peptide moiety. The three NRPS genes of the thanapeptin gene cluster encode nine, four and nine adenylation (A)-domains, respectively. The prediction of the 22 amino acids activated by these A-domains is shown in Figure 5. The C-terminus of the last gene of the cluster encodes two thioesterase (TE)-domains, indicating termination of the thanapeptin synthesis. Despite the similarity between syringopeptin and thanapeptin (Figure 2), there also is a clear difference in modular organization as syringopeptin is produced from three NRPS modules, with five, five and twelve adenylation domains, respectively. Comparison with the *P. corrugata* CFBP5454 draft genome sequence shows that the corpeptin gene cluster sequence is incomplete and scattered over contigs. Nevertheless, for the annotated parts, protein identities with the SH-C52 NRPSs ThpA and ThpC ranged between 75 and 95%, whereas for ThpB identities ranged between 60 and 80%.

Using MALDI and live colony NanoDESI mass spectrometry, a specific group of ions was detected for which the parent mass ranged from 2082 to 2150 Da. Comparing the tandem MS spectra of this set of ions indicated these are related peptides, probably produced by the same NRPS gene cluster. Usage of the peptidogenomics approach (Kersten et al., 2011) on the tandem MS data of these ions (Supplementary Data) revealed that they can be linked to the predicted thanapeptin peptide sequence, and thus to the thanapeptin NRPS gene cluster. MS analysis of the extracts from the thanapeptin mutants indeed showed that the production of all ions of the putative thanapeptin group were absent (data not shown). Further analysis of the tandem MS data of the subsequently purified ion of 2120 Da was in agreement with the *in silico* predicted amino acid sequence for thanapeptin. In addition, tandem MS data provided evidence for cyclization, and resolved the identity of the lipid moiety of thanapeptin (Figure 5 and Supplementary Presentation 1).

**Figure 6:**
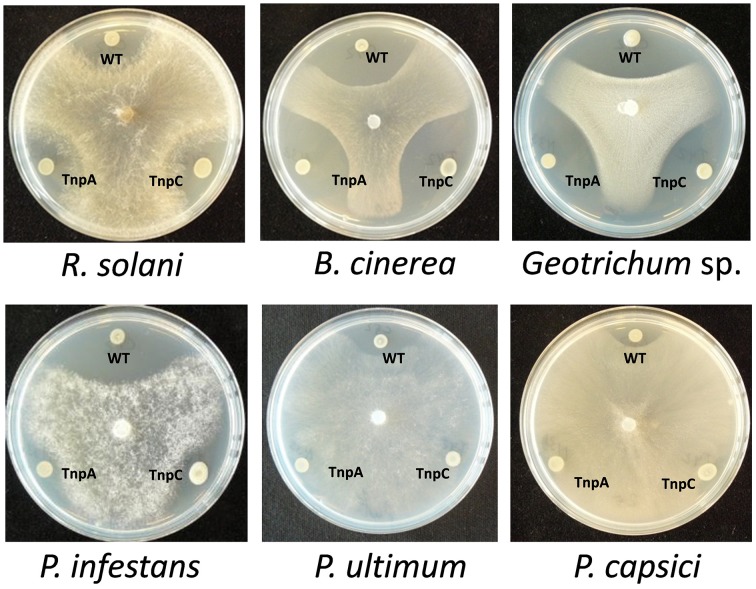
**Antagonistic activity of ***Pseudomonas*** sp. SH-C52**. The wild-type (WT) and the thanapeptin mutants strains, Tn-*tnpA* (TnpA) and Tn-*tnpC* (TnpC) were tested in dual culture assays for activity against fungal plant pathogens **(upper panel)** and oomycete plant pathogens **(lower panel)**.

**Figure 7:**
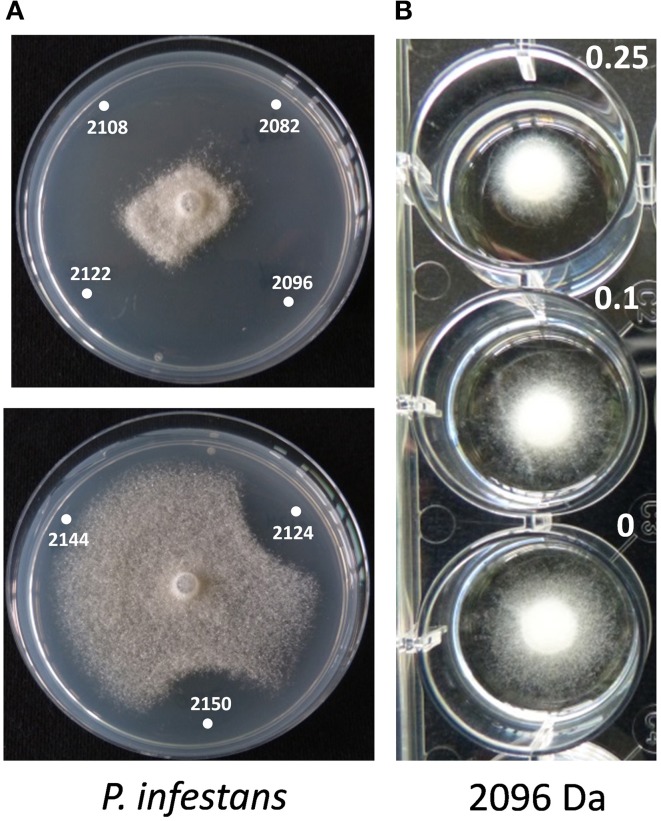
**Activity of thanapeptin derivatives against ***Phytophthora infestans***. (A)** Activity of thanapeptin tested on plate. Numbers indicated in the pictures are the parent mass of the derivate tested in Da. **(B)** For the derivate of 2096 Da the growth-inhibitory activity was tested in liquid broth, at different concentrations, indicated in the picture in μg/ml.

The role of the thanapeptin gene cluster in the activity of SH-C52 against fungi, oomycetes and bacteria was studied by comparing wild-type strain SH-C52 and two independent mutants, each with a single transposon insertion in the thanapeptin NRPS gene cluster. The two mutants lost their antagonistic activity against oomycete pathogens, whereas they still had activity against fungi (Figure 6) and bacteria (data not shown). From the wild-type strain, thanapeptin derivatives were purified and seven derivatives were tested for activity against the oomycete *P. infestans*. Substantial differences in anti-oomycete activity were observed between the compound derivatives, with the strongest activity for those with the lowest mass, i.e., compounds with the masses 2082, 2096, 2108, and 2122 Da (Figure 7A). In addition, two derivatives with strong activity, 2096 and 2122 Da, were also tested against the oomycete pathogens *Saprolegnia parasitica* and *P. ultimum*. Similar results were obtained as in the assays with *P. infestans*, with the strongest activity for the derivative with a mass of 2096 Da, and slightly lower activity for the derivative with a mass of 2122 Da (data not shown). Subsequently, the activity of the derivative with a mass of 2096 Da was tested at different concentrations against the three oomycete pathogens in a liquid broth. For *P. infestans* (Figure 7), a clear reduction in mycelial growth was observed at a concentration of 0.25 μg/ml. For syringopeptin or corpeptin, no anti-oomycete activity has been reported to date (Vassilev et al., 1996; Emanuele et al., 1998). No apparent antifungal activity or activity against Gram-negative bacteria was observed for any of the thanapeptin derivatives, whereas activity was observed against the Gram-positive bacterium *B. megaterium* (data not shown), which is in line with results observed previously for syringopeptin and corpeptin (Vassilev et al., 1996; Emanuele et al., 1998).

## Discussion

*Pseudomonas* sp. SH-C52 was initially studied for its role in the natural soil suppressiveness against the fungal pathogen *R. solani* (Mendes et al., 2011). Here we show by both genome and metabolomic analyses that this plant-associated bacterium has a much greater genomic capacity for the production of secondary metabolites. We showed that *Pseudomonas* sp. SH-C52 can be placed within subgroup I of P. fluorescens species (Redondo-Nieto et al., 2012) as a separate clade together with *P. corrugata* and *P mediterranea*. *P. corrugata* strains have been reported for biological control, for bioremediation and for the production of a range of biomolecules. In contrast, *P. corrugata* strains have also been reported as plant pathogenic bacteria (Catara, 2007). Indeed, the sequenced strain *P. corrugata* CFBP5454 was isolated because of its pathogenic properties (Licciardello et al., 2007). In contrast, *Pseudomonas* sp. SH-C52 was isolated because of its plant-beneficial and antagonistic properties (Mendes et al., 2011). For *P. corrugata*, regulation of cormycin and corpeptin production is under the control of an *N*-acyl-homoserine-lactone quorum sensing system (Licciardello et al., 2012), for which the genes are conserved in strain SH-C52 (data not shown). For both, the quorum sensing system is located down-stream of the thana/corpeptin gene clusters. The different origin of these strains suggests that structurally identical secondary metabolites can have diverse functions, depending on the niche where they are produced. For instance they can fight off pathogen threats for certain plant niches, as is the case for thanamycin in protecting sugar beet seedlings, or are advantageous to occupy certain niches, as is the case for cormycin during infection of plants.

In addition to thanamycin and thanapeptin, *Pseudomonas* sp. SH-C52 produces a third LP with antimicrobial activity. Previously, brabantamides were shown to be active against specific Gram-positive bacteria (Reder-Christ et al., 2012; Schmidt et al., 2014). Here we report that brabantamide A also has activity against oomycetes. Interestingly, this compound has received considerable attention for the development of the synthetic phospholipase inhibitor darapladib, which is tested for treatment of atherosclerosis (Johnston et al., 2013). This highlights the importance of identifying novel LPs for the development of new medicines. In this respect it is interesting to note that in the genome of *Pseudomonas* sp. SH-C52 a putative fourth LP-NRPS gene cluster is present, with a yet unknown structure and function. Also an *mgoA*-like NRPS gene was identified in the genome of SH-C52. Although the *mgo*-operon was shown to be essential for mangotoxin production in mango-pathogenic *P. syringae* strains (Arrebola et al., 2012), it was later demonstrated that the biosynthesis of mangotoxin occurs by means of the *mbo*-operon (Carrion et al., 2012). This suggests that MgoA likely has a regulatory function (Carrion et al., 2014). An orthologous gene cluster was shown to be essential for insect pathogenicity in *P. entomophila*, however, no pathogenicity factor or compound has yet been linked to this phenotype (Vallet-Gely et al., 2010). The *mgo*-operons are not only present in pathogenic bacteria, but are widespread among *Pseudomonas* species, including species from the *P. fluorescens* clade (Loper et al., 2012). Since the product from MgoA, or the *mgo*-operon, has not been deduced, the function of the *mgo*-operon remains an intriguing question. The Mgo-product may act as a *Pseudomonas* specific switch for secondary metabolite production or as a genus- or species-specific regulator involved in different functions.

The production of secondary metabolites often comes at the expense of primary metabolism (Ruiz et al., 2010). Therefore, production needs to be tightly regulated in a temporal and spatial manner. *Pseudomonas* sp. SH-C52 has the ability to produce at least three and likely four structurally different LPs, each with structural variants. LPs have diverse natural functions in motility, biofilm formation, antibiosis, defense or virulence (Raaijmakers and Mazzola, 2012). These functions have to be strictly regulated and cannot be performed all at once. Some LPs are produced at the same time but may have different roles. This is the case for two LPs produced by *Pseudomonas* CMR12a, i.e., sessilin and orfamide that are produced together but involved in respectively biofilm formation and swarming. Regulation takes place by the fact that sessilin hampers the release of orfamide by co-precipitating with orfamide (D'Aes et al., 2014). Although we do see that thanamycin and thanapeptin can be produced simultaneously by *Pseudomonas* SH-C52 (Figure 5), we do not see the simultaneous production of the other two LPs. This suggests dissimilar regulatory pathways for the production of these LPs, possibly with negative and positive feedback loops. Three of the LPs produced by SH-C52 have antimicrobial activity *in vitro*, but it remains to be studied if these LPs also have antimicrobial activity *in vivo*. In this respect, future studies will focus on the identification of regulatory pathways and identification of specific extracellular cues that trigger the production of the structurally diverse LPs in SH-C52 in interactions with diverse organisms encountered in the rhizosphere environment.

## Author contributions

MV designed and performed microbiological, molecular and chemical experiments, performed *in silico* genome analysis and drafted the manuscript. HM performed growth inhibition assays with *P. infestans* and experiments to test phospholipase activity. JW designed chemical experiments, helped in chemical analysis, and performed the chemistry and analysis on the thanapeptin structure, ED assisted in microbiological and molecular experiments. RM was involved in acquiring and analysing genome information. YS and HG purified brabantamide A for phospholipase and antimicrobial assays. PD designed chemical experiments and was involved in chemical imaging. JR supervised the work and was involved in the experimental design. All authors contributed to the writing of the manuscript and approved submission.

### Conflict of interest statement

The authors declare that the research was conducted in the absence of any commercial or financial relationships that could be construed as a potential conflict of interest.
