# Supplementary material for: Genome mining and metabolic profiling of the rhizosphere bacterium Pseudomonas sp. SH-C52 for antimicrobial compounds
Source: Front Microbiol. 2015 Jul 7;6:693. doi: 10.3389/fmicb.2015.00693 (PMC4493835; doi:10.3389/fmicb.2015.00693)
Supplement: Supplementary file 1 [file Table1.DOCX]

Table S1: Thanapeptin gene cluster sequencing primers

| Primer | sequence (5'-3') |
| --- | --- |
| 1_F_thanapeptin | agtgcctcaagctgcacata |
| 1_R_thanapeptin | gatgctggacgtgttcctg |
| 2_F_thanapeptin | atgtaggcaatccgatccac |
| 2_R_thanapeptin | aactatcgcgagctcaatgg |
| 4_F_thanapeptin | gaccgactgctcgatcaac |
| 4_R_thanapeptin | gtacacctcgggttctaccg |
| 6_F_thanapeptin | caccagactgcgtttgagc |
| 6_R_thanapeptin | aactacccgctgatgctctc |
| 7_F_thanapeptin | ggccaacaacgagtgtcc |
| 7_R_thanapeptin | gtgagatcgaagccaagctc |
| 8_F_thanapeptin | cgctagtcaacggtgcaaag |
| 8_R_thanapeptin | gcattcagcttcacggtcat |
| 9_F_thanapeptin | gaccagcacagtaccgaaca |
| 9_R_thanapeptin | actgcatcagcgtttcgac |
| 10_F_thanapeptin | cgcttcgtactcacgggtaa |
| 10_R_thanapeptin | cgcgaagtgatccagacc |
| 11_F_thanapeptin | tcaaggtcaatgggtaattgg |
| 11_R_thanapeptin | ggcagtggcatcgaagag |
| 12_F_thanapeptin | acacggaaaccacggatct |
| 12_R_thanapeptin | ctcaaatgtgctgccgtcta |
| 13_F_thanapeptin | acggttcaggtagccgttc |
| 13_R_thanapeptin | gccttcgatgccagtactct |
| 14_F_thanapeptin | ctccagcatgtaggcaatcc |
| 14_R_thanapeptin | tgaggtgctcagtggtgaag |
| 15_F_thanapeptin | cgcttccgattgagtgaaat |
| 15_R_thanapeptin | tgtgcaacctctacggtcct |
| 16_F_thanapeptin | gtagatctcacccggtacgc |
| 16_R_thanapeptin | atgggttgttcgaggatcag |
| 17_F_thanapeptin | gtaaccacgggcaacacag |
| 17_R_thanapeptin | ctgcgacctgaaggatgtc |
| 18_F_thanapeptin | cgtcagtcgatcctcaaacc |
| 18_R_thanapeptin | gagcacactgcatgttcgac |
| 4_F_R2_thanapeptin | tcctgacgaccggacaag |
| 4_R_R2_thanapeptin | ggtgaaatcgaccgcatagt |
| 2_F_R2_thanapeptin | catcgacaaagtccgacaga |
| 2_R_R2_thanapeptin | acgaaacgacggatatcgag |
| 1_F_R2_thanapeptin | aggtgcgcaagatgtcgt |
| 1_R_R2_thanapeptin | ggcggggtatgtagatgct |
| peptin_R3_after_Fp1 | CACCGGCTGCTTGTGTTCA |
| peptin_R3_after_Rp1 | TTGCTGGCGATCCTCAAG |
| peptin_R3_middle_Fp2 | AACGTCTTCACGGGCCAGT |
| peptin_R3_middle_Rp2 | CCCTGTCGAAAACCCTGT |
| peptin_R3_before_Fp3 | GAAGTGATCGTGACGACC |
| peptin_R3_before_Rp3 | CTGATGGAGGCACTGTTC |
| fp_R4_s1 | TCAGGCTGACAGCCAACAAG |
| rp_R4_s1 | GGTGGAAACGCTCTTCGA |
| fp_R4_s2 | TTTCGATTTCGCCTTGCG |
| rp_R4_s2 | TTTGCACCGTTGACCAGC |
| fp_R4_s3 | ATGCGGGCGTTTGGAACAG |
| rp_R4_s3 | AGGCAGGTGCTGCAGGACTT |

note: Equal numbers indicate primers belonging tot the same primer pair. F and R indicates forward or reverse primer, respectively.
