# Supplementary material for: Genome mining and metabolic profiling of the rhizosphere bacterium Pseudomonas sp. SH-C52 for antimicrobial compounds
Source: Front Microbiol. 2015 Jul 7;6:693. doi: 10.3389/fmicb.2015.00693 (PMC4493835; doi:10.3389/fmicb.2015.00693)
Supplement: Supplementary file 2 [file Presentation1.PPTX]

## Slide 1
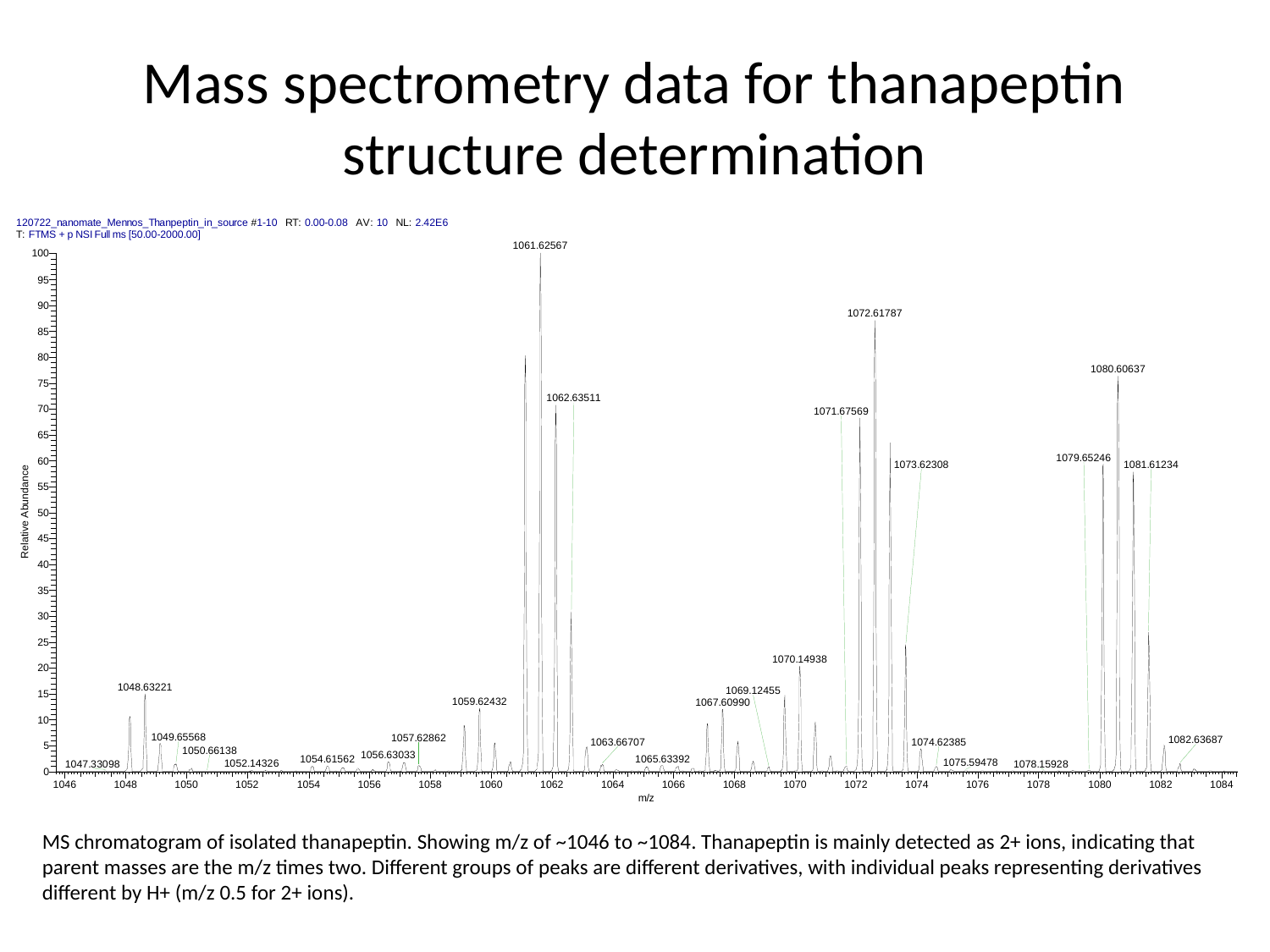

# Mass spectrometry data for thanapeptin structure determination
MS chromatogram of isolated thanapeptin. Showing m/z of ~1046 to ~1084. Thanapeptin is mainly detected as 2+ ions, indicating that parent masses are the m/z times two. Different groups of peaks are different derivatives, with individual peaks representing derivatives different by H+ (m/z 0.5 for 2+ ions).

## Slide 2
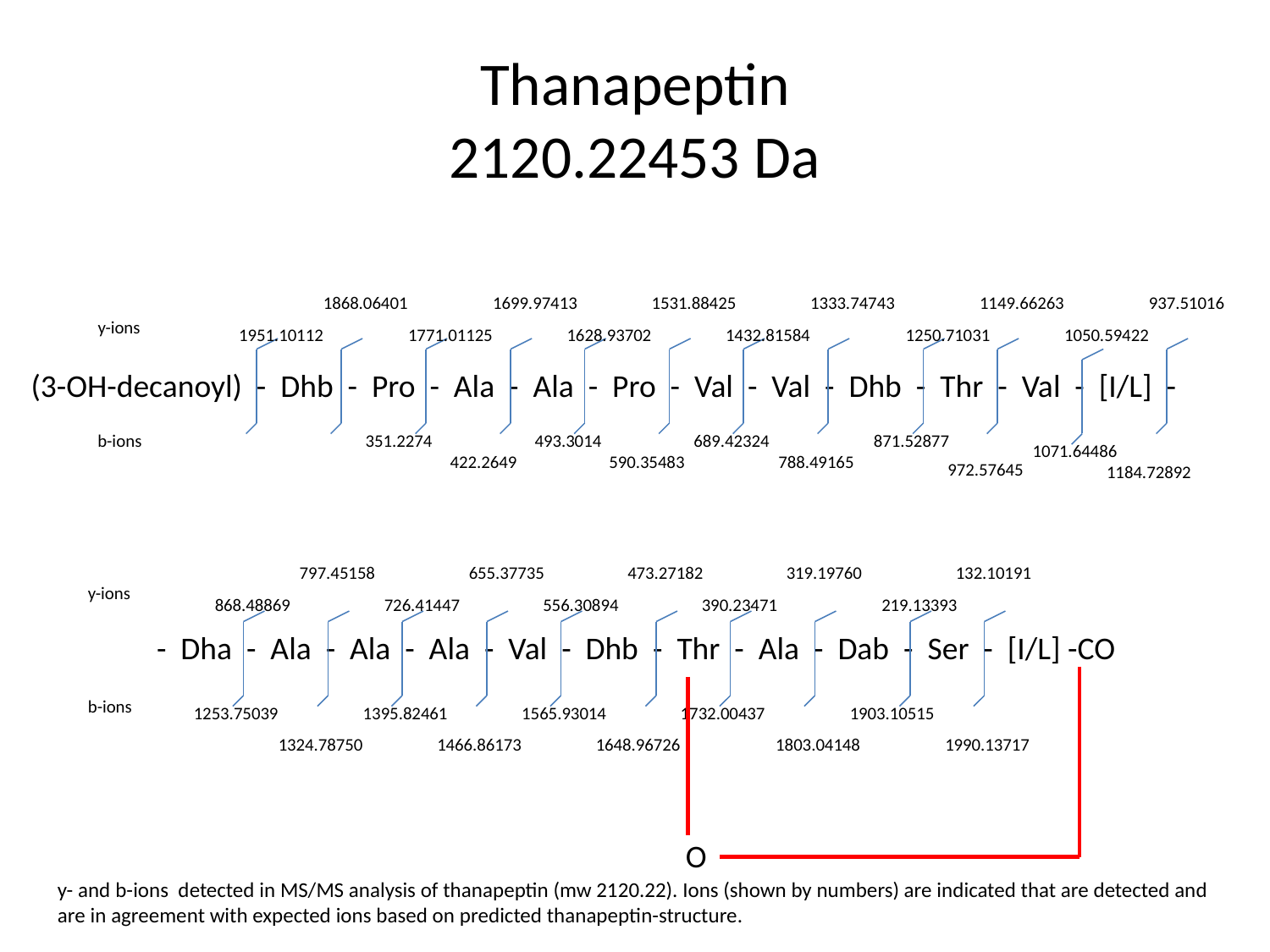

# Thanapeptin 2120.22453 Da
1868.06401
1699.97413
1531.88425
1333.74743
1149.66263
937.51016
y-ions
1951.10112
1771.01125
1628.93702
1432.81584
1250.71031
1050.59422
(3-OH-decanoyl) - Dhb - Pro - Ala - Ala - Pro - Val - Val - Dhb - Thr - Val - [I/L] -
b-ions
351.2274
493.3014
689.42324
871.52877
1071.64486
422.2649
590.35483
788.49165
972.57645
1184.72892
797.45158
655.37735
473.27182
319.19760
132.10191
y-ions
868.48869
726.41447
556.30894
390.23471
219.13393
- Dha - Ala - Ala - Ala - Val - Dhb - Thr - Ala - Dab - Ser - [I/L] -CO
b-ions
1253.75039
1395.82461
1565.93014
1732.00437
1903.10515
1324.78750
1466.86173
1648.96726
1803.04148
1990.13717
O
y- and b-ions detected in MS/MS analysis of thanapeptin (mw 2120.22). Ions (shown by numbers) are indicated that are detected and are in agreement with expected ions based on predicted thanapeptin-structure.

## Slide 3
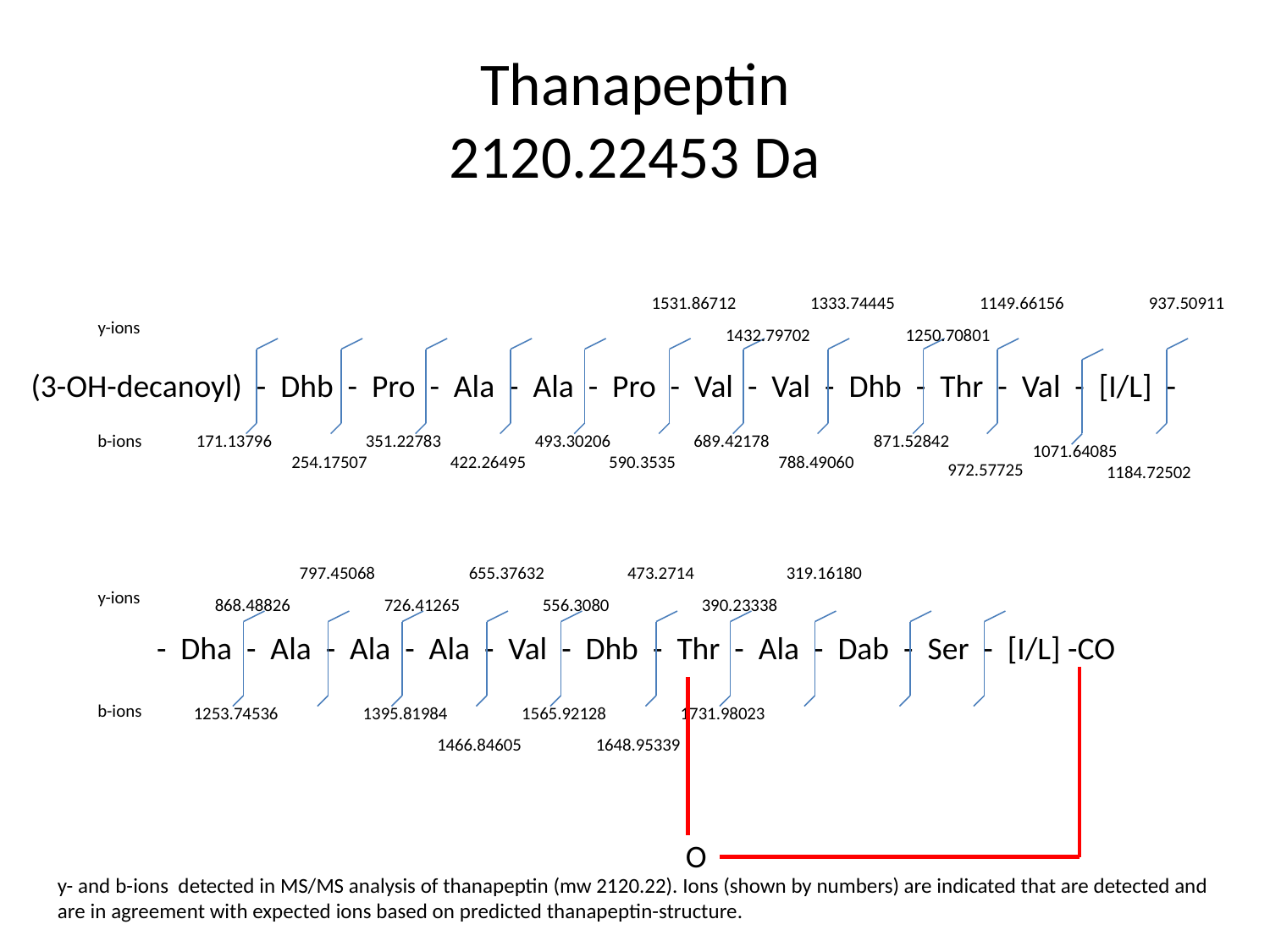

# Thanapeptin 2120.22453 Da
1531.86712
1333.74445
1149.66156
937.50911
y-ions
1432.79702
1250.70801
(3-OH-decanoyl) - Dhb - Pro - Ala - Ala - Pro - Val - Val - Dhb - Thr - Val - [I/L] -
b-ions
171.13796
351.22783
493.30206
689.42178
871.52842
1071.64085
254.17507
422.26495
590.3535
788.49060
972.57725
1184.72502
797.45068
655.37632
473.2714
319.16180
y-ions
868.48826
726.41265
556.3080
390.23338
- Dha - Ala - Ala - Ala - Val - Dhb - Thr - Ala - Dab - Ser - [I/L] -CO
b-ions
1253.74536
1395.81984
1565.92128
1731.98023
1466.84605
1648.95339
O
y- and b-ions detected in MS/MS analysis of thanapeptin (mw 2120.22). Ions (shown by numbers) are indicated that are detected and are in agreement with expected ions based on predicted thanapeptin-structure.

## Slide 4
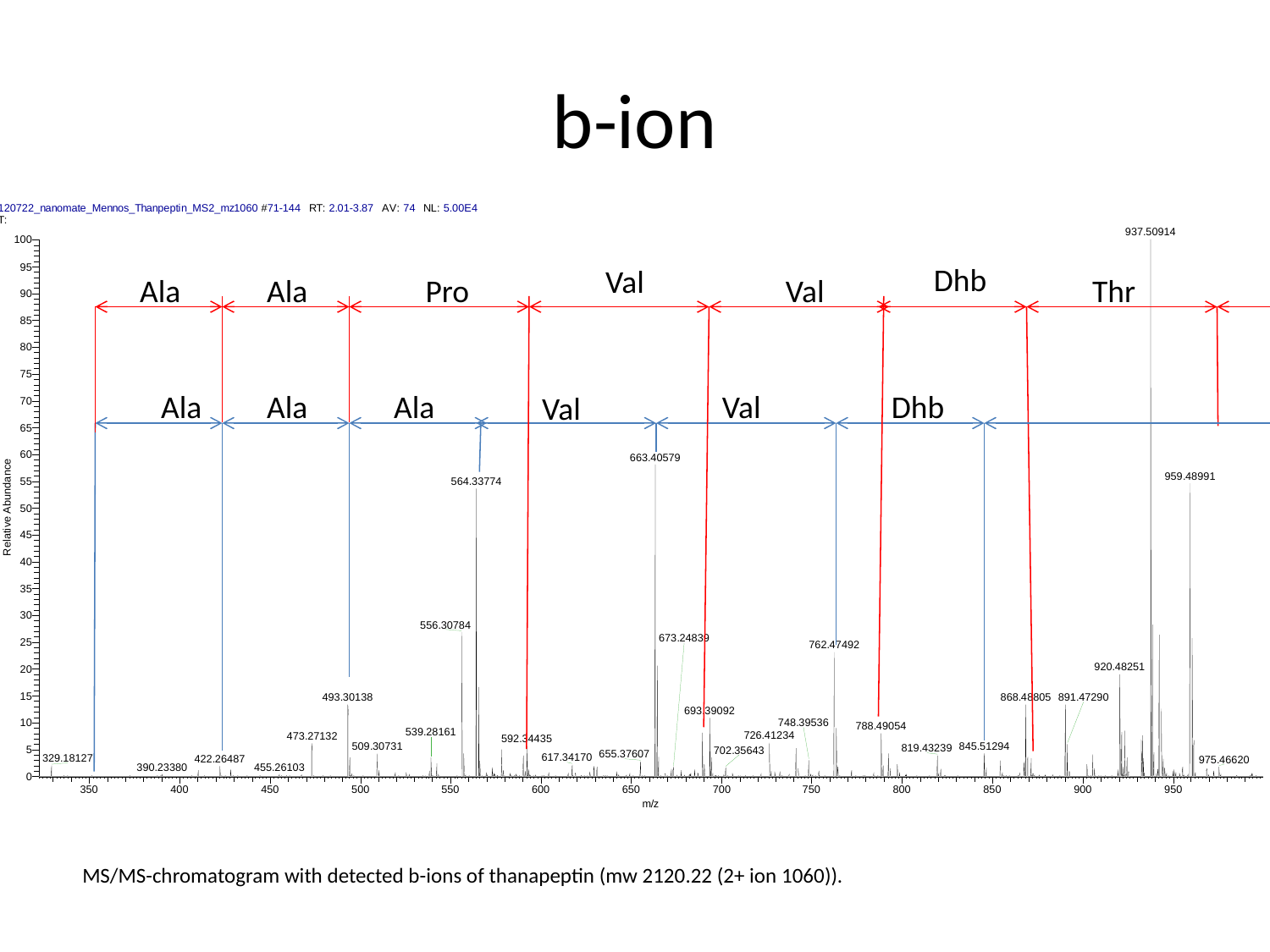

# b-ion
Dhb
Val
Ala
Ala
Pro
Val
Thr
Ala
Ala
Ala
Val
Dhb
Val
MS/MS-chromatogram with detected b-ions of thanapeptin (mw 2120.22 (2+ ion 1060)).

## Slide 5
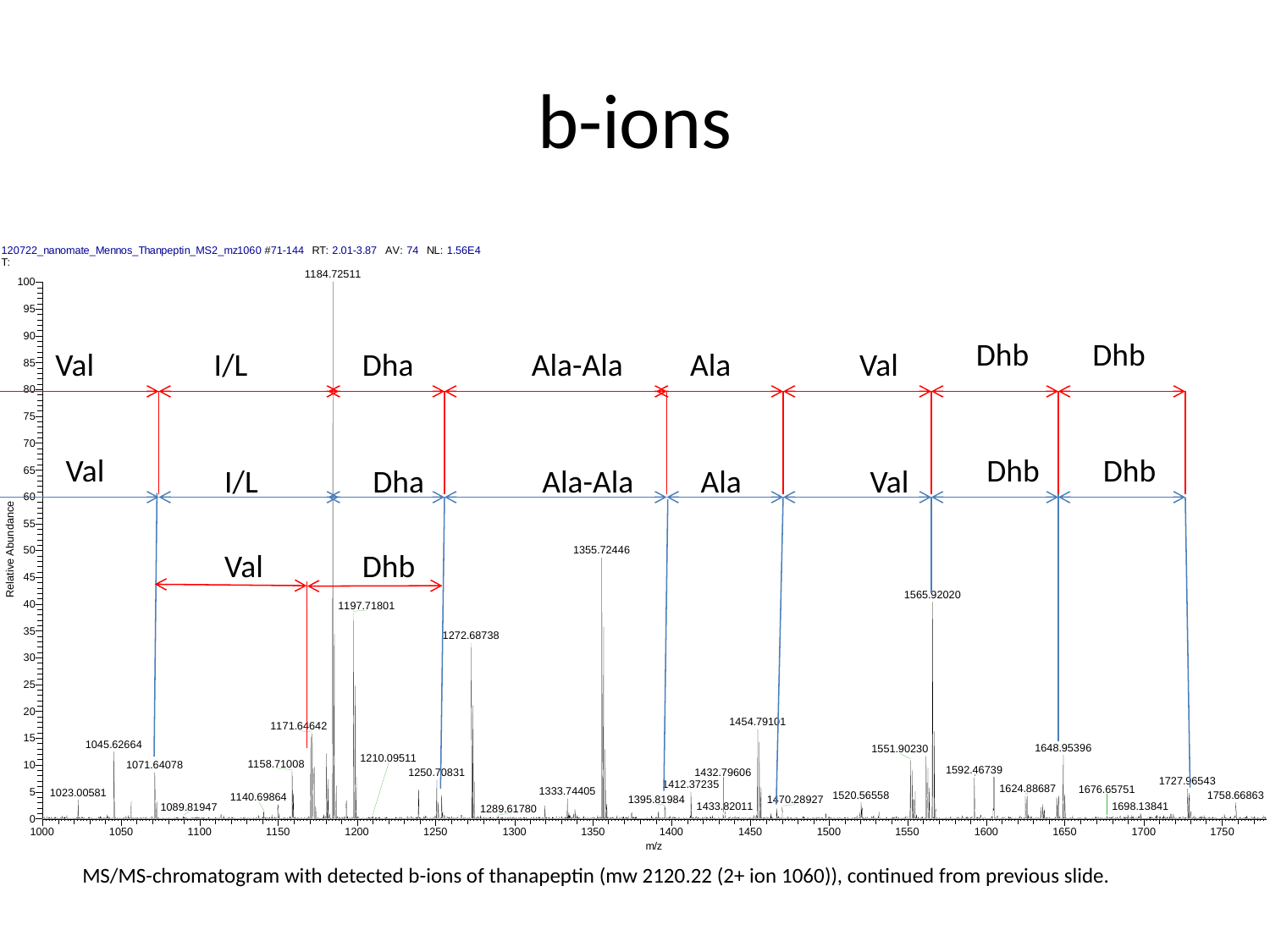

# b-ions
Dhb
Dhb
Val
I/L
Dha
Ala-Ala
Ala
Val
Val
Dhb
Dhb
I/L
Dha
Ala-Ala
Ala
Val
Val
Dhb
MS/MS-chromatogram with detected b-ions of thanapeptin (mw 2120.22 (2+ ion 1060)), continued from previous slide.

## Slide 6
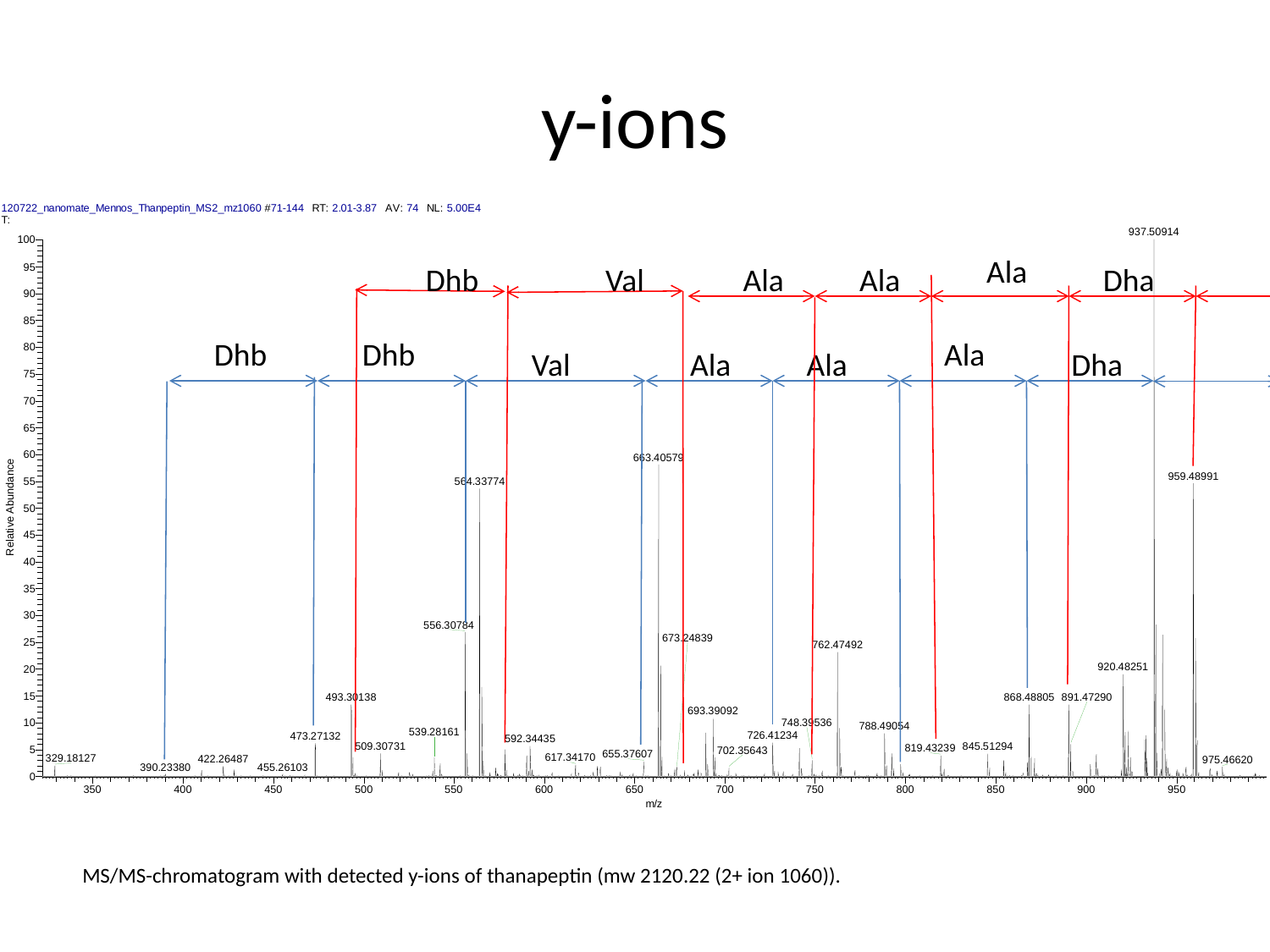

# y-ions
Ala
Dhb
Val
Ala
Ala
Dha
Dhb
Dhb
Ala
Val
Ala
Ala
Dha
MS/MS-chromatogram with detected y-ions of thanapeptin (mw 2120.22 (2+ ion 1060)).

## Slide 7
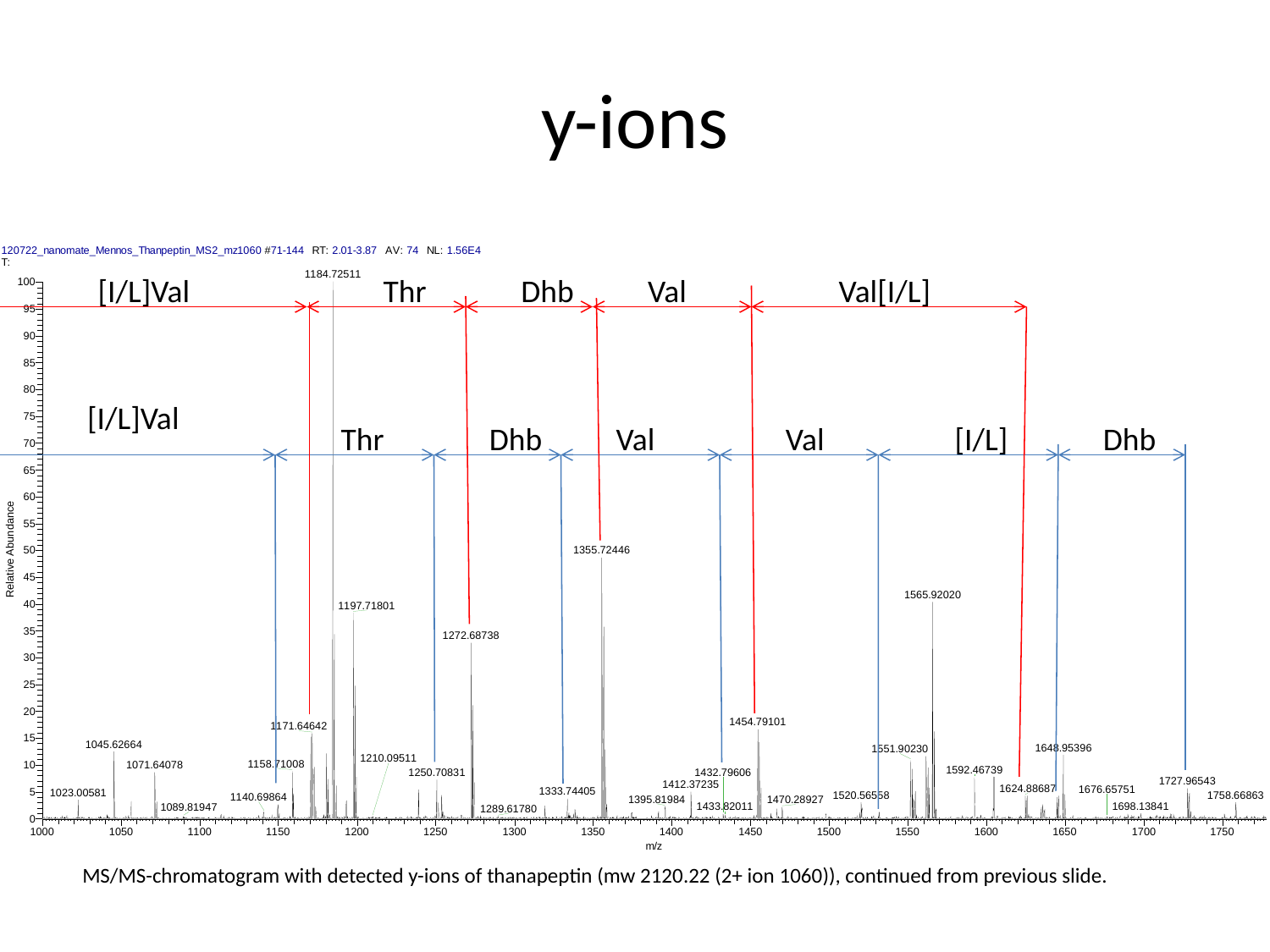

# y-ions
[I/L]Val
Thr
Dhb
Val
Val[I/L]
[I/L]Val
Thr
Dhb
Val
Val
[I/L]
Dhb
MS/MS-chromatogram with detected y-ions of thanapeptin (mw 2120.22 (2+ ion 1060)), continued from previous slide.
